# The telomerase gene polymorphisms, but not telomere length, increase susceptibility to primary glomerulonephritis/end stage renal diseases in females

**DOI:** 10.1186/s12967-020-02347-3

**Published:** 2020-05-04

**Authors:** Qing Sun, Junli Liu, Guanghui Cheng, Mingkai Dai, Jiaxi Liu, Zhenqiang Qi, Jingjie Zhao, Wei Li, Feng Kong, Gang Liu, Magnus Björkholm, Dawei Xu

**Affiliations:** 1 Central Research Laboratory, Shandong University Second Hospital, Jinan, 250035 People’s Republic of China; 2 Laboratory for Molecular Diagnostics, Shandong University Second Hospital, Jinan, 250035 People’s Republic of China; 3 Department of Nephrology, Affiliated Hospital of Shandong University of Traditional Chinese Medicine, Jinan, 250013 People’s Republic of China; 4 Nephrology Research Institute of Shandong University, Shandong University Second Hospital, Jinan, 250035 People’s Republic of China; 5 Department of Medicine, Center for Molecular Medicine and Bioclinicum, Karolinska Institutet, Karolinska University Hospital Solna, SE-171 76 Stockholm, Sweden

**Keywords:** CKD, Genotypes, Glomerulonephritis, Single nucleotide polymorphism, Telomerase, Telomere

## Abstract

**Background:**

Primary glomerulonephritis (GN) is the leading cause of chronic kidney disease (CKD) and frequently progresses into end stage renal diseases (ESRDs). Shorter leukocyte telomere length (LTL) has been implicated in the CKD susceptibility and diminished kidney function, however, it is unclear whether the variants in *telomerase* genes contribute to risk to GN/CKD/ESRD. Here we address this issue by determining their association with the genetic variants of rs12696304 at the *telomerase RNA component (TERC)* and rs2736100 at the *telomerase reverse transcriptase (TERT)* loci.

**Methods:**

The study includes 769 patients (243 primary GN-derived CKD and 526 ESRD cases) and sex-/age-matched healthy controls. Genomic DNA was extracted from peripheral blood of both controls and patients. Genotyping of rs12696304 and rs2736100 variants was carried out using PCR-based assays. Leukocyte telomere length (LTL) was determined using quantitative PCR (qPCR).

**Results:**

A significantly higher frequency of TERC rs12696304 G allele was observed in patients and associated with increased disease risk (C vs G: OR = 1.334, 95% CI 1.112–1.586, *P* = 0.001; CC + GC vs GG: OR = 1.334, 95% CI 1.122–1.586, *P* = 0.001). Further analyses showed that such significant differences were only present between female controls and patients (C vs G: OR = 1.483, 95% CI 1.140–1.929, *P* = 0.003; CC + GC vs CC: OR = 1.692, 95% CI 1.202–2.383, *P* = 0.003), but not males. There were no differences in rs2736100 variants between controls and patients, but female ESRD patients carried significantly higher C allele frequencies than did female controls (A vs C: OR = 1.306, 95% CI 1.005–1.698, *P* = 0.046; AA vs CC: OR = 1.781, 95% CI 1.033–3.070, *P* = 0.037). There was no difference in LTL between controls and patients.

**Conclusions:**

Our results reveal that the *TERC* rs12696304 and *TERT* rs2736100 polymorphisms, but not LTL per se, contribute to GN/CDK/ESRD risk.

## Background

The prevalence of chronic kidney disease (CKD) has significantly increased in the last decades, affecting more than 10% of the adult population worldwide [1]. In general, primary glomerulonephritis (GN) is a leading cause of CKD and frequently progresses into end stage renal disease (ESRD) [1, 2], which has been a great burden to the public health care system [3]. The precise pathogenesis of primary GN/CKD is unclear, but the host-genetic background is implicated in the disease onset and progression [4–6]. Epidemiological studies showed striking geographic and ethnical variations [2, 4]. Consistently, recent genome-wide association studies (GWAS) and genetic analyses revealed a panel of genetic variants associated with the disease susceptibility and/or complications [4–9].

Human linear chromosomes terminate with telomeric TTAGGG repeat sequences essential for genomic stability and integrity [10–12]. Telomeric DNA is synthesized by telomerase, an RNA dependent DNA polymerase with telomerase reverse transcriptase (TERT) and telomerase RNA template (TERC) as its core components [11–13]. Most human somatic cells express no or low levels of telomerase activity, which, together with “the end-replication problem”, results in progressive telomere shortening with cellular divisions [10–12]. When telomere becomes too short (dysfunctional) to protect chromosomes, the DNA damage response is activated, thereby triggering the permanent growth arrest of cells so-called replicative senescence. Thus, telomere erosion serves as a mitotic clock recording the number of cell divisions and limiting their life-span, and is widely accepted as a biomarker for aging and age-related conditions [10–12, 14]. Indeed, short leukocyte telomere length (LTL) has been shown to be associated with cancer, increased mortality, cardiovascular disorders, stroke, diabetes, and other age-related disorders [10, 15]. Similarly, shorter LTL-carriers were reported to display significantly decreased glomerular filtration rate while increased urinary albumin-creatinine ratio [16–18]. Carrero et al. and others further observed that CKD or ESRD patients had accelerated telomere attrition coupled with increased mortality [19, 20]. These findings suggest that shorter LTL is likely associated with CKD prevalence/occurrence or declining kidney function. However, there are published reports showing the lack of such an association [17]. In addition, because adverse environmental factors are capable of driving premature telomere erosion, the causal relationship between shortened LTL and GN/CKD/ESRD remains unclear.

There exist multiple single nucleotide polymorphisms (SNPs) in the *telomerase* genes and some of them are significantly associated with LTL in the general populations, and risk of disorders as described above [21–29]. However, the relationship between these SNPs and GN/CKD/ESRD risk has not been explored yet so far. Moreover, unlike LTL, these germline variants are not affected by environmental elements. In the present study, we thus sought to determine whether rs12696304 at the *TERC* and rs2736100 at the *TERT* loci, two well characterized LTL-related SNPs, contribute to susceptibility to primary GN/CKD/ESRD.

## Methods

### Study populations

The case–control individuals include 515 healthy controls and 769 primary GN/CKD/ESRD patients and they were all Han Chinese. Primary non-end stage GN/CDK patients (n = 243) were recruited from the out-patient service/department or ward, at Shandong University Second Hospital, Shandong University Qilu Hospital, Shandong Provincial Hospital and the Affiliated Hospital of Shandong University of Traditional Chinese Medicine, between June 2016 and Jan. 2018. ESRD patients, developed from primary GN, were included from Shandong University Second Hospital, between Jan. 2012 and Dec. 2017 and blood was collected before they underwent kidney transplantation. Five hundred and fifteen unrelated healthy controls who were age- and sex-matched to cases were recruited from the Physical Examination Center of Shandong University Second Hospital. All of the included controls had normal kidney function. The study was approved by the Ethics Review Committee of Shandong University Second Hospital and informed consent was obtained from all participants.

### DNA extraction and genotyping of the TERC rs12696304 and TERT rs2736100 variants

Genomic DNA was extracted from peripheral blood cells using TIANGEN DNA extraction kits. The *TERC* rs12696304(C/G) and *TERT* rs2736100 (A/C) genotyping was carried out using pre-designed TaqMan SNP genotyping assay kits on an ABI 7500 Life Tech (Applied Biosystems), as described [30]. Both positive and negative controls were included in all assays and the running condition was as followed: 95 °C for 5 min, followed by 40 cycles of 92 °C for 15 s and 60 °C for 30 s.

### LTL assay

Genomic DNA was isolated from peripheral blood cells as described above and LTL was assessed using real-time PCR as previously described [31, 32]. Briefly, 2 ng of DNA were used for each PCR reaction. The primer sequences for human telomere (Tel 1b and Tel 2b) and β-globin (HBG3 and HBG4) were: Tel1b: 5′-CGGTTTGTTTGGGTTTGGGT-TTGGGTTTGGGTTTGGGTT-3′; Tel2b: 5′-GGCTTGCCTTACCCTTACCCTTACCC-TTACCCTTACCCT-3′; HBG3: 5′-TGTGCTGGCCCATCACTTTG-3′, and HBG4: 5′-ACCAGCCA-CCACTTTCTGATAGG-3′. T/HBG values were determined using the formula T/S = 2−ΔCt, where ΔCt = average Ct_telomere_ − average Ct_β-globin_. The T/S ratio was arbitrarily expressed as LTL. Age-adjusted LTL for each control and patient was done by subtracting the subject’s linear predicted LTL from the observed one.

### Statistical analyses

The evaluation of distribution differences of selected variables and alleles of the *TERC* rs12696304 and *TERT* rs2736100 between GN/CKD/ESRD patients and healthy controls were done using χ2 test. Hardy–Weinberg equilibrium of the genotype distribution among the controls and cases were tested by a goodness-of-fit χ2 test. Unconditional univariate and multivariate logistic regression analyses were used to estimate Odd ratios (ORs) for risk of GN/CKD/ESRD and their 95% confidence intervals (CIs). The LTL difference between patients and healthy controls was assessed using Student *T* test. All the tests were computed using SPSS17.0 software. For comparison of rs12696304 and rs2736100 alleles and genotypes between healthy controls and GN/CKD/ESRD patients, *P* values of < 0.05 were considered as statistically significant.

## Results

### Characteristics of study subjects

A total of 769 patients with primary GN/CKD/ESRD were included in the present study. Age and sex distributions are shown in Table 1. Five hundred and fifteen unrelated healthy adults used as controls were age- and sex-matched (Table 1). Both controls and patients were genotyped for *TERC* rs12696304 and *TERT* rs2736100 variants. LTL was assessed in 327 controls and 592 patients.

**Table 1:** Characteristics of primary GN/CKD/ESRD patients & healthy controls

	Primary GN		Healthy controls
	CKD (non-end stage)	ESRDs	
Number	243	526	515
Age (year)			
Range	13–81	15–83	15–86
Mean ± SD	43 ± 14	46 ± 11	46 ± 12
Gender			
Male	133	301	289
Female	110	225	226

*GN* Glomerulonephritis, *CKD* chronic kidney disease, *ESRD* end-stage renal disease

### The association between the rs12696304 G allele or GG genotype and susceptibility to primary GN/CKD/ESRD

We first determined rs12696304 and rs2736100 allele/genotype distribution in both controls and patients with GN/CKD/ESRD. Genotyping was successfully performed on DNA from all 515 controls and 757/769 patients for rs2736100, and all 515 controls and 759/769 patients for rs12696304, respectively. The results, summarized in Table 2, showed a significantly increased frequency of rs12696304 G allele in the patient group compared to that in controls (C vs G: OR = 1.465, 95% CI 1.170–1.834, *P* = 0.001; Table 2). Similarly, a higher frequency of the rs12696304 GG genotype was observed in the patient group (CC + GC vs GG: OR = 1.334, 95% CI 1.122–1.586, *P* = 0.001; Table 2). Because our previous studies showed that telomerase gene variant-associated disease susceptibility was gender-dependent [32, 33], we compared males and females separately. The significant difference was only confined to female controls and patients (C vs G: OR = 1.483, 95% CI 1.140–1.929, *P* = 0.003; CC + GC vs CC: OR = 1.692, 95% CI 1.202–2.383, *P* = 0.003; Table 2), whereas not seen in the male groups (Table 2). These findings thus suggest that the rs12696304 G allele and GG genotype are significantly associated with primary GN/CKD/ESRD risk in females.

**Table 2:** Genotyping results of rs12696304 and rs2736100 in controls and patients with GN/CKD/ESRD

	Controls (M + F)	Patients (M + F)	Odds ratio (95% CI)	*P* value	Controls (M only)	Patients (M only)	Odds ratio (95% CI)	*P* value	Controls (F only)	Patients (F only)	Odds ratio (95% CI)	*P* value
rs12696304 (N)	515 (100%)	759 (100%)			289 (100%)	434 (100%)			226(100%)	326(100%)		
CC	56 (10.9)	64 (8.4)	1.0 (ref.)		32 (11.1)	40 (9.2)	1.0 (ref.)		24 (10.6)	24 (7.4)	1.0 (ref.)	
GC	223 (43.3)	275 (36.2)	1.079 (0.723–1.609)	0.709	119 (41.2)	157 (36.3)	1.055 (0.626–1.779)	0.840	104 (46.0)	118 (36.2)	1.135 (0.608–2.118)	0.692
GG	236 (45.8)	420 (55.4)	1.557 (1.052–2.306)	*0.026*	138 (47.7)	236 (54.5)	1.368 (0.821–2.279)	0.227	98 (43.4)	184 (56.4)	1.878 (1.013–3.478)	*0.043*
CC + GC	279 (54.2)	339 (44.6)	1.0 (ref.)		151 (52.3)	197 (45.5)	1.0 (ref.)		128 (56.6)	142 (43.6)	1.0 (ref.)	
GG	236 (45.8)	420 (55.4)	1.465 (1.170–1.834)	*0.001*	138 (47.7)	236 (54.5)	1.311 (0.973–1.767)	0.089	98 (43.4)	184 (56.4)	1.692 (1.202–2.383)	*0.003*
C	335 (32.5)	403 (26.5)	1.0 (ref.)		183 (31.7)	237 (27.4)	1.0 (ref.)		152 (33.6)	166 (25.5)	1.0 (ref.)	
G	695 (67.5)	1115 (73.5)	1.334 (1.122–1.586)	*0.001*	395 (68.3)	629 (72.6)	1.230 (0.977–1.548)	0.078	300 (66.4)	486 (74.5)	1.483 (1.140–1.929)	*0.003*
rs2736100 (N)	515 (100%)	757 (100%)			289 (100%)	426 (100%)			226 (100%)	331 (100%)		
AA	158 (30.7)	222 (29.3)	1.0 (ref.)		93 (32.2)	138 (32.4)	1.0 (ref.)		65 (28.8)	84 (25.4)	1.0 (ref.)	
AC	257 (49.9)	364 (48.1)	1.008 (0.778–1.306)	0.952	136 (47.1)	201 (47.2)	0.996 (0.708–1.401)	0.982	121 (53.5)	163 (49.2)	1.042 (0.699–1.555)	*0.839*
CC	100 (19.4)	171 (22.6)	1.217 (0.884–1.676)	0.229	60 (20.7)	87 (20.4)	0.977 (0.641–1.489)	0.914	40 (17.7)	84 (25.4)	1.625 (0.989–2.670)	*0.055*
A	573 (55.6)	808 (53.4)	1.0 (ref.)		322 (55.7)	477 (56.0)	1.0 (ref.)		251 (55.5)	331 (50.0)	1.0 (ref.)	
C	457 (44.4)	706 (46.6)	1.098 (0.934–1.284)	0.261	256 (44.3)	375 (44.0)	0.989 (0.799–1.223)	0.918	201 (44.5)	331 (50.0)	1.249 (0.982–1.588)	*0.070*

*GN* Glomerulonephritis, *CKD* chronic kidney disease, *ESRD* end-stage renal disease, *M* male, *F* female, *CI* confidence interval; italic P value: statistically significant

Because certain protective variants may accumulate during disease progression from GN/CKD to ESRD, we divided the patients into two categories: non-end stage GN/CKD and ESRD, and then analyzed their association with rs12696304 variants separately. For CKD cases, both G allele and GG genotype were significantly higher compared to those in controls (C vs G: OR = 1.555, 95% CI 1.215–1.990, *P* = 0.0001; CC + GC vs CC: OR = 1.634, 95% CI 1.201–2.234, *P* = 0.002; Table 3). Again, we only observed such differences between female controls and patients (C vs G: OR = 1.816, 95% CI 1.248–2.641, *P* = 0.002; CC + GC vs CC: OR = 1.959, 95% CI 1.233–3.114, *P* = 0.006; Table 3). There was no difference in the male groups (Table 3).

**Table 3:** The association of rs12696304 variants with primary GN/CKD risk

	Controls (M + F)	Patients (M + F)	Odds ratio (95% CI)	*P* value	Controls (M only)	Patients (M only)	Odds ratio (95% CI)	*P* value	Controls (F only)	Patients (F only)	Odds ratio (95% CI)	*P* value
rs12696304(N)	515 (100%)	243 (100%)			289 (100%)	133 (100%)			226(100%)	110(100%)		
CC	56 (10.9)	13 (5.3)	1.0 (ref.)		32 (11.1)	9 (6.7)	1.0 (ref.)		24 (10.6)	4 (3.6)	1.0 (ref.)	
GC	223 (43.3)	89 (36.6)	1.719 (0.896–3.298)	0.135	119 (41.2)	49 (36.8)	1.464 (0.651–3.294)	0.465	104 (46.0)	40 (36.4)	2.308 (0.753–7.070)	0.207
GG	236 (45.8)	141 (58.1)	2.574 (1.359–4.873)	*0.004*	138 (47.7)	75 (56.5)	1.932 (0.876–4.263)	0.141	98 (43.4)	66 (60.0)	4.041(1.340–12.181)	*0.015*
CC + GC	279 (54.2)	102 (41.9)	1.0 (ref.)		151 (52.3)	58 (43.5)	1.0 (ref.)		128 (56.6)	44 (40.0)	1.0 (ref.)	
GG	236 (45.8)	141 (58.1)	1.634 (1.201–2.234)	*0.002*	138 (47.7)	75 (56.5)	1.415 (0.936–2.139)	0.122	98 (43.4)	66 (60.0)	1.959 (1.233–3.114)	*0.006*
C	335 (32.5)	115 (23.7)			183 (31.7)	67 (25.2)	1.0 (ref.)		152 (33.6)	48	1.0 (ref.)	
G	695 (67.5)	371 (76.3)	1.555 (1.215–1.990)	*0.0001*	395 (68.3)	199 (74.8)	1.376 (0.992–1.910)	0.067	300 (66.4)	172	1.816 (1.248–2.641)	*0.002*
rs2736100 (N)	515 (100%)	237 (100%)			289 (100%)	129 (100%)			226 (100%)	108 (100%)		
AA	158 (30.7)	66 (27.9)	1.0 (ref.)		93 (32.2)	34 (26.4)	1.0 (ref.)		65 (28.8)	32 (29.6)	1.0 (ref.)	
AC	257 (49.9)	116 (48.9)	1.081 (0.753–1.551)	0.743	136 (47.1)	67 (51.9)	1.348 (0.826–2.199)	0.283	121 (53.5)	49 (45.4)	0.823 (0.481–1.409)	0.566
CC	100 (19.4)	55 (23.2)	1.317 (0.851–2.038)	0.261	60 (20.7)	28 (21.7)	1.276 (0.703–2.317)	0.516	40 (17.7)	27 (25.0)	1.371 (0.719–2.616)	0.428
A	573 (55.6)	248 (52.3)	1.0 (ref.)		322(55.7)	135 (52.3)	1.0 (ref.)		251(55.5)	113 (52.3)	1.0 (ref.)	
C	457 (44.4)	226 (47.7)	1.143 (0.919–1.421)	0.253	256(44.3)	123 (47.7)	1.146 (0.854–1.538)	0.405	201(44.5)	103 (47.7)	1.138 (0.822–1.576)	0.485

*GN* Glomerulonephritis, *CKD* chronic kidney disease, *M* male, *F* female, *CI* confidence interval; italic P value: statistically significant

Similar distribution patterns of rs12696304 SNPs as seen above were also observed in the ESRD group: When a comparison including both males and females was made, the patient group exhibited significantly increased frequencies of G allele and GG genotype (C vs G: OR = 1.245, 95% CI 1.031–1.503, *P* = 0.022; CC + GC vs CC: OR = 1.392, 95% CI 1.089–1.778, *P* = 0.010; Table 4); whereas again a separate analysis showed that the difference was confined to the female groups (Table 4).

**Table 4:** Differences in rs12696304 and rs2736100 genotyping between controls and patients with ESRD

Patients (M + F)	Odds ratio (95% CI)	*P* value	Controls (M only)	Patients (M only)	Odds ratio (95% CI)	*P* value	Controls (F only)	Patients (F only)	Odds ratio (95% CI)	*P* value
516 (100%)			289 (100%)	300 (100%)			226 (100%)	216 (100%)		
51 (9.9)	1.0 (ref.)		32 (11.1)	31 (10.3)	1.0 (ref.)		24 (10.6)	20 (9.3)	1.0 (ref.)	
186 (36.0)	0.916 (0.598–1.403)	0.686	119 (41.2)	108 (36.0)	0.937 (0.536–1.637)	0.819	104 (46.0)	78 (36.1)	0.900(0.464–1.745)	0.755
279 (54.1)	1.298 (0.855–1.970)	0.219	138 (47.7)	161 (53.7)	1.204 (0.699–2.074)	0.502	98 (43.4)	118 (54.6)	1.445 (0.753–2.771)	0.266
237 (45.9)	1.0 (ref.)		151 (52.3)	139 (46.3)	1.0 (ref.)		128 (56.6)	98 (45.4)	1.0 (ref.)	
279 (54.1)	1.392 (1.089–1.778)	*0.010*	138 (47.7)	161 (53.7)	1.267 (0.917–1.752)	0.176	98 (43.4)	118 (54.6)	1.573 (1.080–2.289)	*0.023*
288 (27.9)	1.0 (ref.)		183 (31.7)	170 (28.3)	1.0 (ref.)		152 (33.6)	118 (27.3)	1.0 (ref.)	
744 (72.1)	1.245 (1.031–1.503)	*0.022*	395 (68.3)	430 (71.7)	1.172 (0.913–1.504)	0.213	300 (66.4)	314 (72.7)	1.348 (1.011–1.798)	*0.042*
520 (100%)			289 (100%)	297 (100%)			226 (100%)	223 (100%)		
156 (30.0)	1.0 (ref.)		93 (32.2)	104 (35.0)	1.0 (ref.)		65 (28.8)	52 (23.3)	1.0 (ref.)	
248 (47.7)	0.977 (0.737–1.295)	0.873	136 (47.1)	134 (45.1)	0.881 (0.610–1.273)	0.500	121 (53.5)	114 (51.1)	1.178 (0.754–1.839)	0.472
116 (22.3)	1.175 (0.830–1.662)	0.363	60 (20.7)	59 (19.9)	0.879 (0.558–1.387)	0.580	40 (17.7)	57 (25.6)	1.781 (1.033–3.070)	*0.037*
560 (53.8)	1.0 (ref.)		322 (55.7)	342 (57.6)	1.0 (ref.)		251 (55.5)	218 (48.9)	1.0 (ref.)	
480 (46.2)	1.075 (0.904–1.278)	0.415	256 (44.3)	252 (42.4)	0.927 (0.736–1.168)	0.519	201 (44.5)	228 (51.1)	1.306 (1.005–1.698)	*0.046*

*ESRD* End-stage renal disease, *M* male, *F* female, *CI* confidence interval; italic P value: statistically significant

### The association between the rs2376100 C allele or CC genotype and susceptibility to ESRD

The *TERT* rs2736100 genotyping was performed in these same controls and patients, and the obtained allele and genotype distribution is shown in Table 2. There was no significant difference in allele or genotype frequencies between controls and patients, or between males and females. However, the analysis of the ESRD subgroup revealed significantly increased frequencies of rs2736100 C allele and CC genotype in female patients compared to their matched healthy controls (A vs C: OR = 1.306, 95% CI 1.005–1.698, *P* = 0.046; AA vs CC: OR = 1.781, 95% CI 1.033–3.070, *P* = 0.037; Table 4).

### LTL in healthy controls and patients

**Fig. 1:**
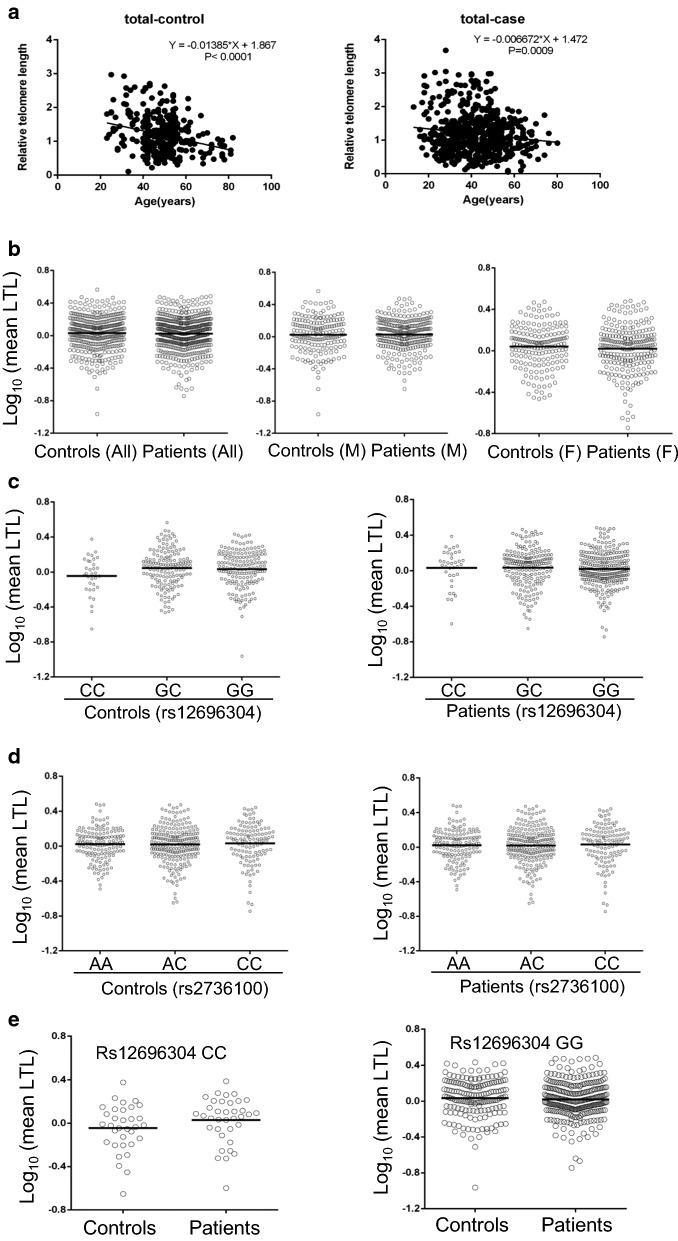
Leukocyte telomere length (LTL) in controls and patients with GN/CKD/ESRD. LTL was assessed using qPCR as described in Methods. **a** The negative correlation between LTL and age in both controls and patients. **b** No differences in LTL between controls and patients. Left: All controls and patients; Middle and right: Male and female controls and patients, respectively. **c** No differences in LTL among different genotypes of rs12696304 in controls (left) and patients (right). **d** Lack of differences in LTL among different genotypes of rs2736100 in controls (left) and patients (right). **e** Lack of association of LTL with rs12696304 CC or GG genotypes between controls and patients. Left and right panels: CC and GG genotypes, respectively

LTL was assessed in 327 controls and 592 patients. In both controls and patients, LTL was negatively correlated with age (P < 0.001) (Fig. 1a). LTL, as assessed using qPCR, was 1.187 ± 0.539 and 1.189 ± 0.593 (mean ± SD) for controls and patients, respectively, which did not differ significantly (*P* = 0.967) (Fig. 1b). Because females in general harbour longer LTL than men, and our results showed an association of telomerase gene variants with in female patients, we further compared LTL between controls and patients according to sex. A total of 159 male controls had LTL 1.160 ± 0.5359, while 363 male patients had LTL 1.193 ± 0.6013 (*P* = 0.967) (Fig. 1c). In females, a slightly shorter LTL was observed in patients (n = 229) compared to that in controls (n = 168) (1.182 ± 0.580 vs 1.213 ± 0.542, however, the difference did not reach a significant level (*P* = 0.590).

As *TERC* rs12696304 and *TERT* rs2736100 variants are both correlated with LTL [21, 22, 24–28, 33, 34], we compared LTL among different genotypes of controls and patients. LTL was 1.055 ± 0.456, 1.210 ± 0.570 and 1.204 ± 0.521 for control CC-, GC- and GG-carriers, respectively, and while 1.129 ± 0.525, 1.230 ± 0.628 and 1.167 ± 0.574 for patient CC-, GC- and GG-carriers, respectively (Fig. 1d). There were no differences among the three different genotypes of both controls and patients, or between controls and patients with the same genotype (Fig. 1e). The comparison of LTL among rs2736100 variants similarly showed the lack of any association between controls and patients irrespective of sex.

## Discussion

Numerous studies have revealed an intimate association between the variants of the *TERT* or *TERC* gene and susceptibility to cancer, aging-associated disorders and many other pathological conditions [15, 21–28, 34, 35], however, their relationship with primary GN/CKD/ESRD has never been explored. In the present study, we investigated the influence of rs2736100 and rs12696304 variants on primary GN/CKD/ESRD risk, and the obtained results demonstrate significantly higher frequencies of the rs12696304 G allele and GG genotype in patients than in healthy controls, which indicate that the *TERC* rs12696304 G allele serves as a biomarker to GN/CKD/ESRD risk. We further observed that such susceptibility occurred only in females with the G allele/GG genotype. The rs2736100 variants are in general not associated GN/CKD risk, but the proportion of female ESRD C allele-carriers was significantly higher compared to their matched control counterparts, suggesting its role in CKD progression. Thus, the present findings provide evidence that the *telomerase* gene SNPs contribute to susceptibility to primary GN/CKD/ESRD.

An autoimmune etiology is strongly implicated in the pathogenesis of primary glomerular diseases/CKDs/ESRDs, while telomerase and telomeres have long been established to play an important role in regulating immunological activity [36, 37]. Activation of telomerase via induction of TERT and TERC expression occurs in activated lymphocytes for their proliferation and clonal expansion in response to infectious challenge [36, 37]. Shorter LTL was associated with increased susceptibility to experimentally induced acute upper respiratory infection and clinical illness in adults [38]. Mechanistically, shorter telomeres limit proliferative potentials of immune cells and compromise immune response to pathogens, thereby lowering host resistance to infection [36, 37]. In addition, telomerase insufficiency and shorter TL due to defective TERT and TERC expression were observed in naïve CD4 T cells from patients with rheumatoid arthritis, through which premature senescence of T cell subsets occurred and subsequent autoimmunity was triggered [39, 40]. Moreover, this scenario was also present in other autoimmune disorders [40]. TERT and TERC are two key components of the telomerase enzyme and it is thus conceivable that their genetic variants may affect telomerase activity. Likely, TERT and TERC variants modify risk of GN/CKD/ESRD by influencing the host immune activity.

However, the LTL comparison between healthy controls and GN/CKD/ESRD patients did not reveal a significant difference, which indicates that telomere homeostasis is not substantially impaired. In addition, the rs12696304 G allele-carriers were previously shown to have significantly shorter LTL in both Chinese and western populations [22, 24–26], but we did not find such scenario in either healthy controls or patients, and therefore the association between rs12696304 G allele and increased GN/CKD/ESRD risk is unlikely attributable to telomere length regulation. Telomere lengthening is the canonical function of telomerase, whereas recent studies have demonstrated its multiple properties independently of telomere homeostasis [11, 41–48]. TERC or TERT has been shown to stimulate the activation of NK-κB pathway to promote inflammatory response independently of telomerase-mediated telomere extension [41, 48], while NK-κB is critical to drive CDK development and progression [49]. The rs12696304 G allele may be involved in a host auto-immune response targeting glomerular tissues via TERC-mediated activation of the NF-κB signalling pathway, which calls for further investigations.

It is intriguing that there exists an association between rs12696304 G allele or GG genotype and GN/CKD/ESRD risk only in females. Because estrogen and progesterone are known to activate the transcription of *TERT* and *TERC* genes [50–53], female sex hormones may play a putative part. As described above, impaired TERT or TERC expression in T cells are involved in the pathogenesis of rheumatoid arthritis. There may be a possibility that female hormones interact with the rs12696304 G allele, thereby contributing to dysregulation of TERC and subsequently leading to an auto-immune attack to glomerular tissues. Further studies are required to elucidate this issue.

Interestingly, we notice a significant difference in the rs12696304 variant distribution between Chinese Han and other ethnical populations. The results reported by us and other investigators showed that the CC genotype was restricted to 9 to 11% in the Chinese population [24], while it was present in more than 30% of Caucasians [22, 25, 26]. It has been well documented that the prevalence of primary GN/CKD/ESRD including IgA nephropathy is substantially higher in China than in Europe and North America, and the genotype difference in rs12696304 likely contributes to this difference in incidence patterns [2]. Similarly, we recently observed the different genotype distribution in the TERT rs2736100 between Swedish and Chinese populations [33]. A significantly higher fraction of rs2736100 C, a risk allele associated with myeloproliferative neoplasia (MPN), was seen in Swedish individuals, which is coupled with a higher incidence of MPN (than that in China) [33]. Collectively, different genotype distributions of telomerase SNPs between Eastern and Western countries may contribute to their different susceptibilities to a wide spectrum of diseases including glomerular diseases.

Our findings also showed that the rs2736100 C allele was associated with ESRDs, but not with non-end stage CKDs. It is likely that this allele contributes to CKD progression or ESRD risk. On the other hand, it may play a protective role in the disease evolution from CKD to ESRD, which thus leads to the selective accumulation of ESRD C allele-carriers.

## Conclusions

The results presented here reveal an association between the rs12696304 G allele or GG genotype and susceptibility to primary glomerular diseases including CKD and ESRD in females. The G allele was shown to be correlated with shorter LTL in a number of studies, but we failed to see such difference in LTL between C and G allele-carriers in both controls and patients. Moreover, we further found that the TERT rs2736100 C allele or CC genotype frequency was higher in female ESRD patients but not in those with CKD, and it is currently unclear whether this is due to evolutional selection during disease progression. Premature telomere erosion has been reported in patients with CKD/ESRD, however, LTL differences were not observed between controls and patients here. Collectively, the TERC rs12696304 variant, and likely the TERT rs2736100 polymorphism, but not LTL per se, are associated with an increased risk of primary glomerular diseases. The present findings provide new insights into telomerase biology and glomerular disease etiology, and may be implicated in the precision prevention/intervention of CKD/ESRD.

## Data Availability

The datasets used and/or analyzed during the current study are available from the corresponding author on reasonable request.

## Abbreviations

CKD: Chronic kidney disease
ESRD: End-stage renal disease
GN: Glomerulonephritis
LTL: Leukocyte telomere length
SNP: Single nucleotide polymorphism
TERC: Telomerase RNA component
TERT: Telomerase reverse transcriptase
